# Supplementary material for: Cardamonin Exerts Antitumor Effect on Human Hepatocellular Carcinoma Xenografts in Athymic Nude Mice through Inhibiting NF-κβ Pathway
Source: Biomedicines. 2020 Dec 9;8(12):586. doi: 10.3390/biomedicines8120586 (PMC7764268; doi:10.3390/biomedicines8120586)
Supplement: Supplementary file 1 [file biomedicines-08-00586-s001.pdf]

Supplementary Materials

Cardamonin Exerts Antitumor Effect on Human Hepatocellular Carcinoma Xenografts in Athymic Nude Mice through Inhibiting NF-κβ Pathway

Nassrin Badroon [1,6], Nazia Abdul Majid [1,*], Fouad Saleih R. Al-Suede [2], Mansoureh Nazari V. [2], Nelli Giribabu [3], Amin Malik Shah Abdul Majid [4], Eltayeb E. M. Eid [5], Mohammed Abdullah Alshawsh [6,*]

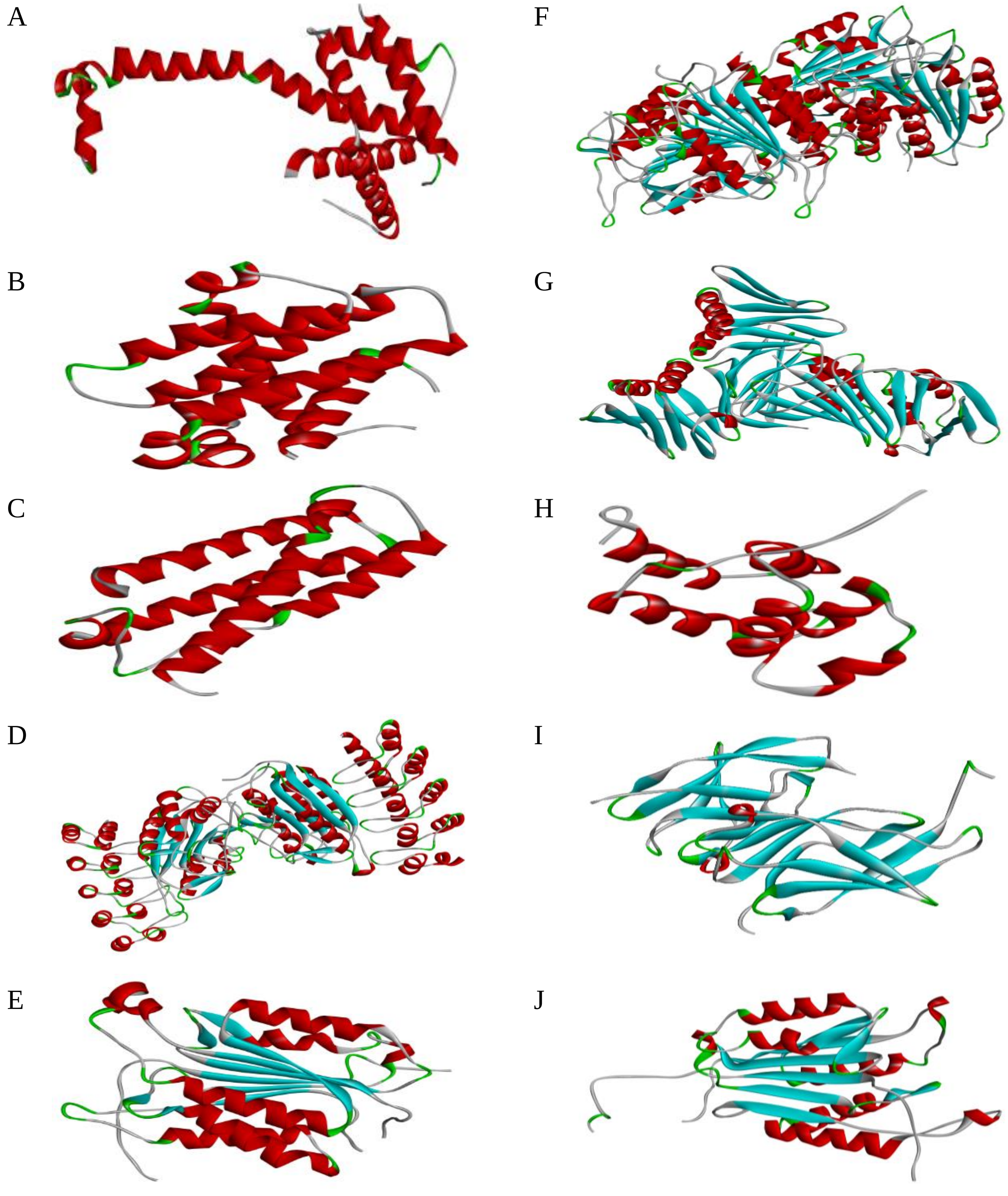

Figure S1. Three dimensional crystal structure of (A) Bax BH3 (4BD6), (B) Bcl-2 (5WHI), (C) Cytochrome C (4CDA), (D) Caspase 7 (4LSZ), (E) Caspase 8 (3KJN), (F) Caspase 9 (1JXQ), (G) PCNA (3WGW), (H) NF-κB (2DBF), (I) NF-κB-p65 (1MY7) and (J) Caspase 3 (3KJF).

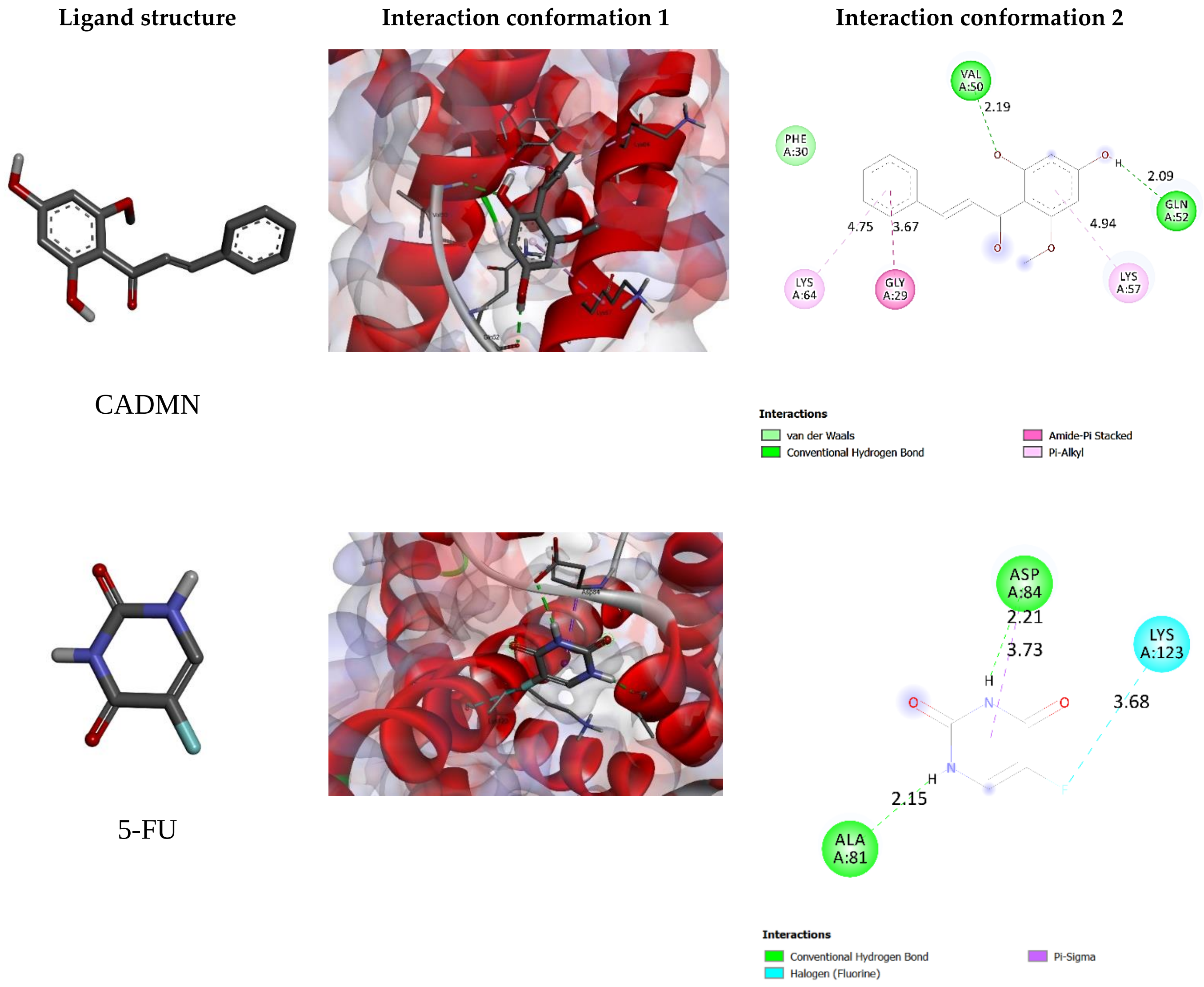

Figure S2. The interactions between Bax BH3 and ligands (CADMN and 5-FU)

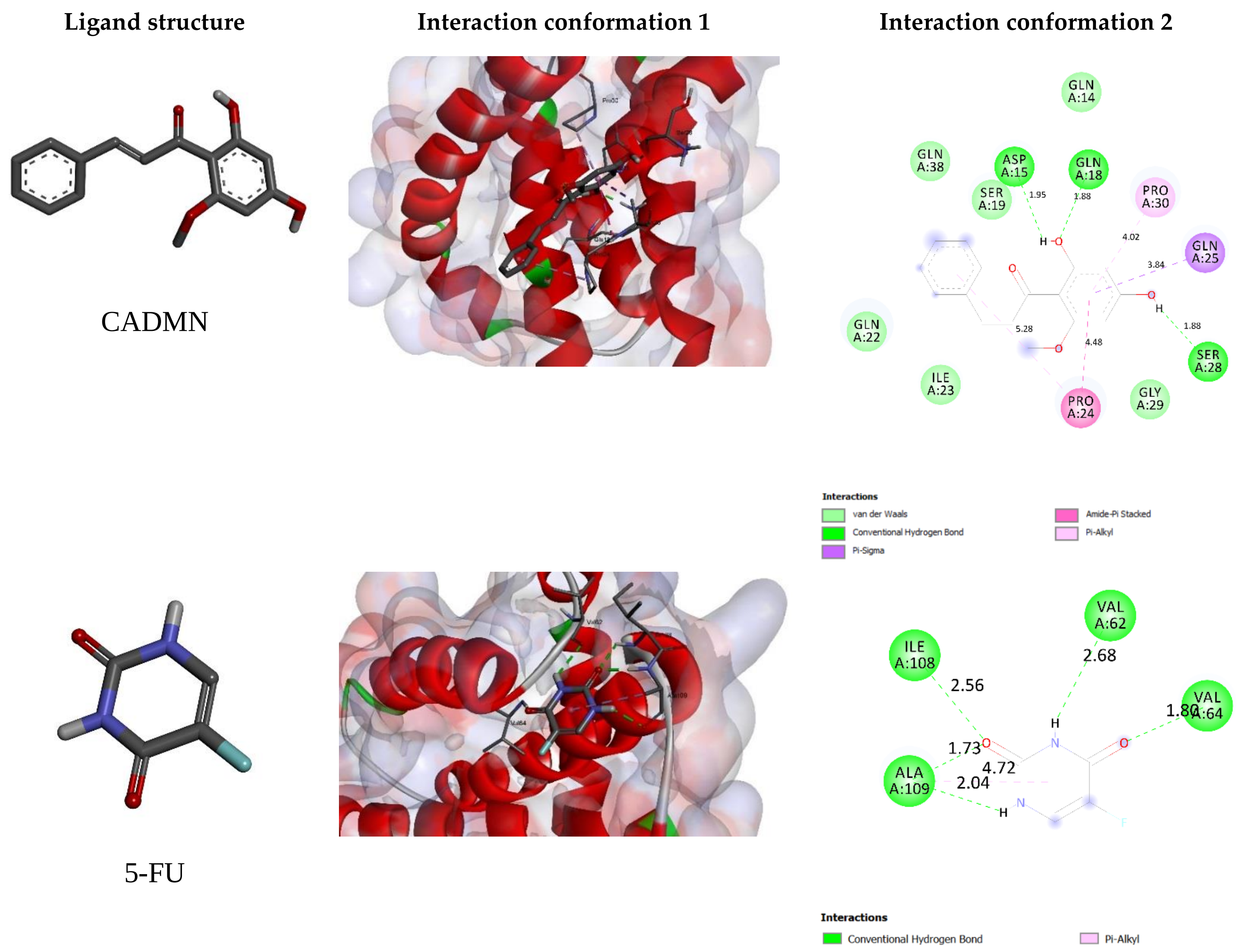

Figure S3. The interactions between Bcl-2 and ligands (CADMN and 5-FU)

Ligand structure | **Interaction conformation 1** | **Interaction conformation 2**

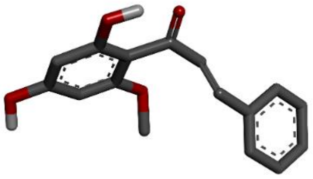

CADMN

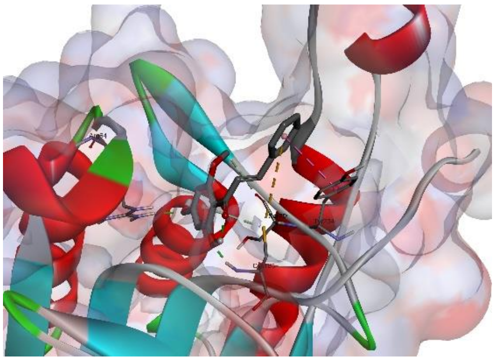

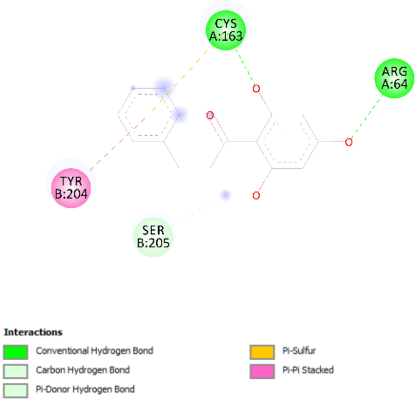

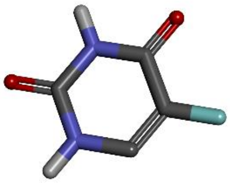

5-FU

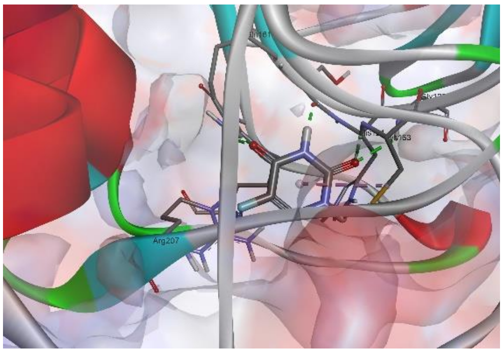

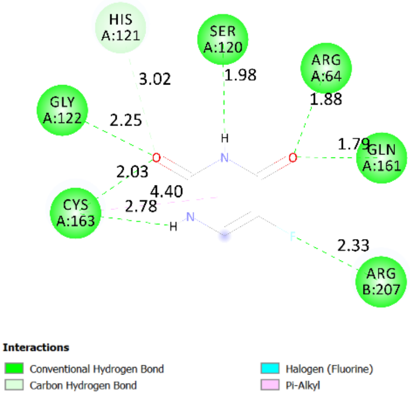

Figure S4. The interactions between caspase 3 and ligands (CADMN and 5-FU

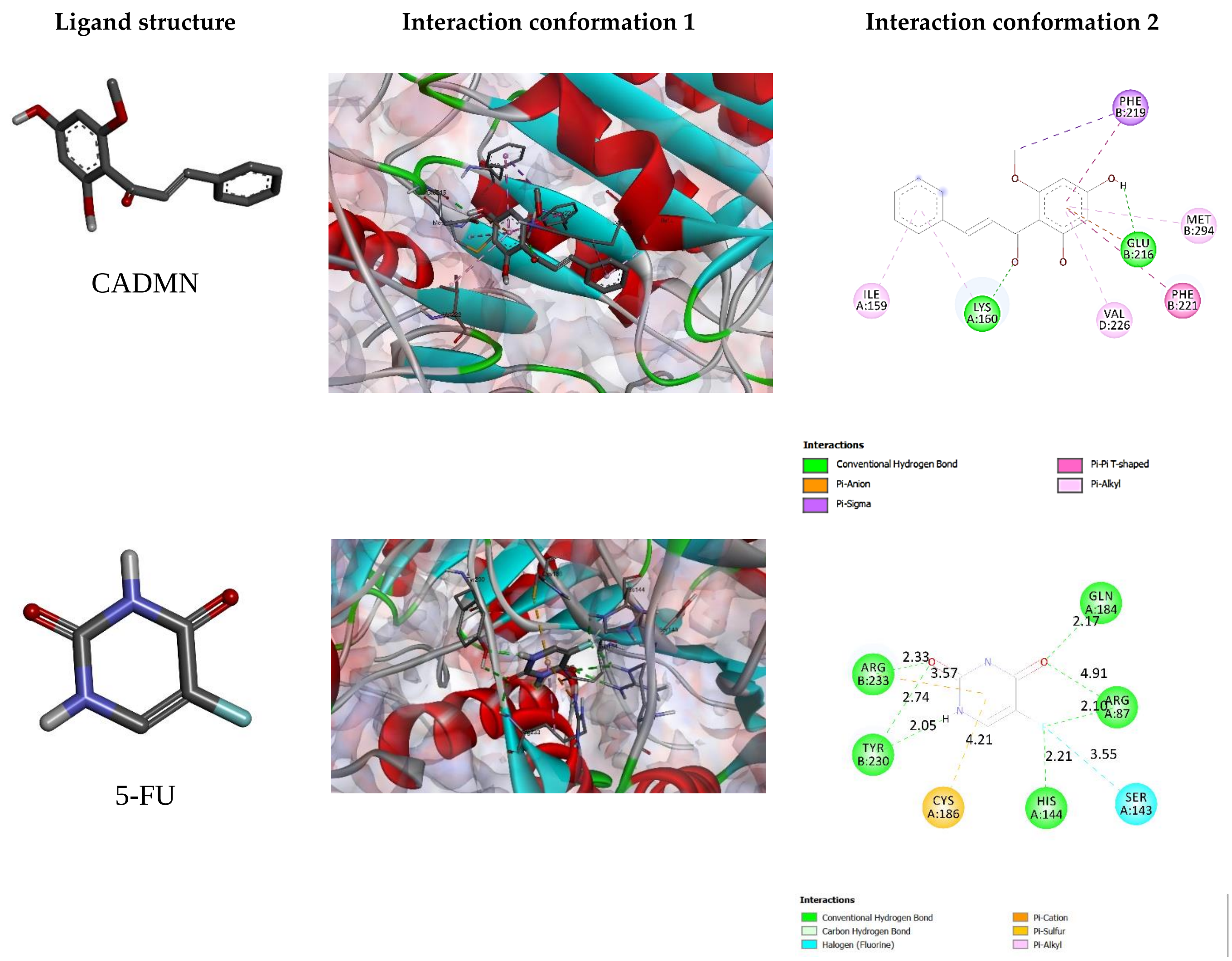

Figure S5. The interactions between caspase 7 and ligands (CADMN and 5-FU)

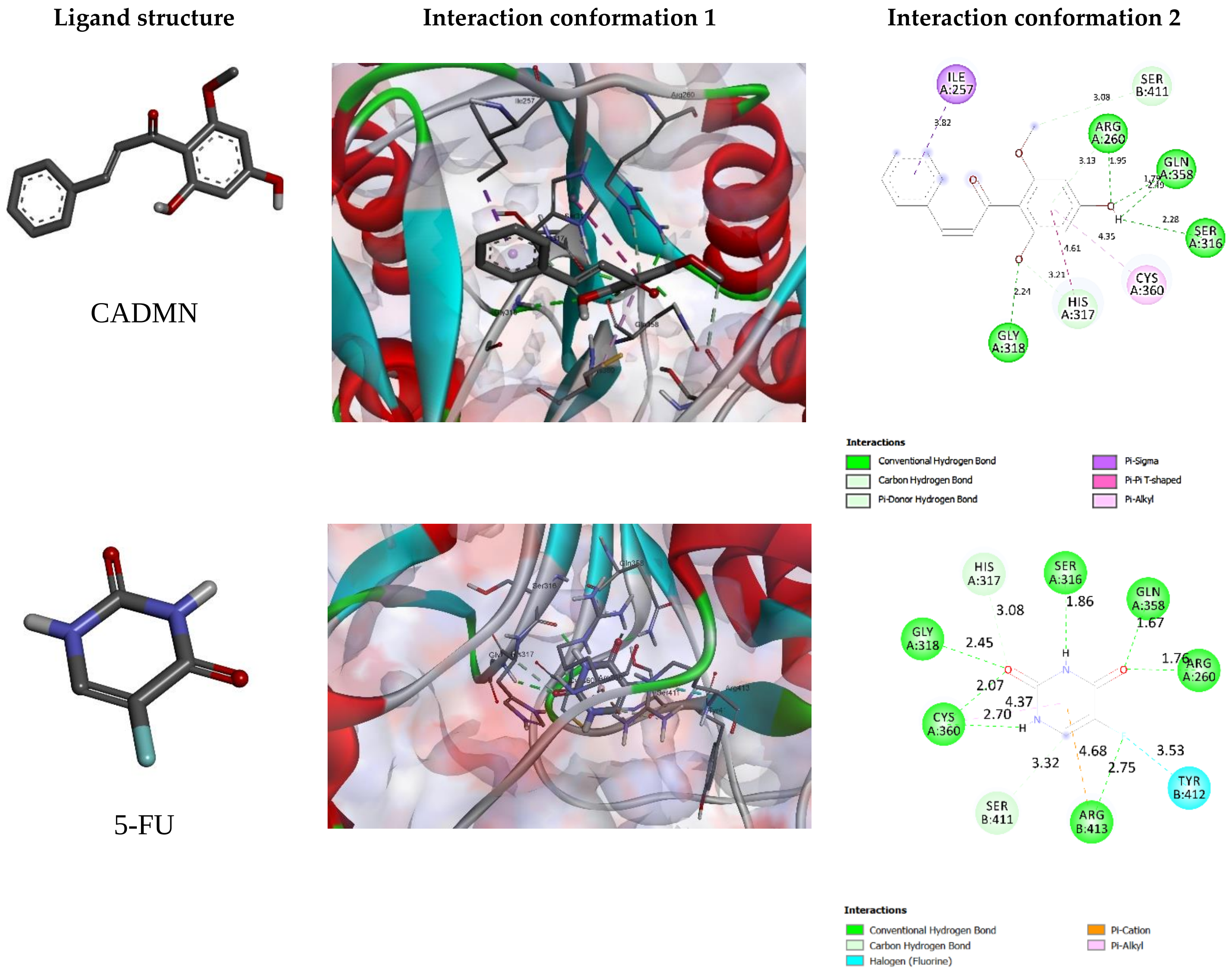

Figure S6. The interactions between caspase 8 and ligands (CADMN and 5-FU)

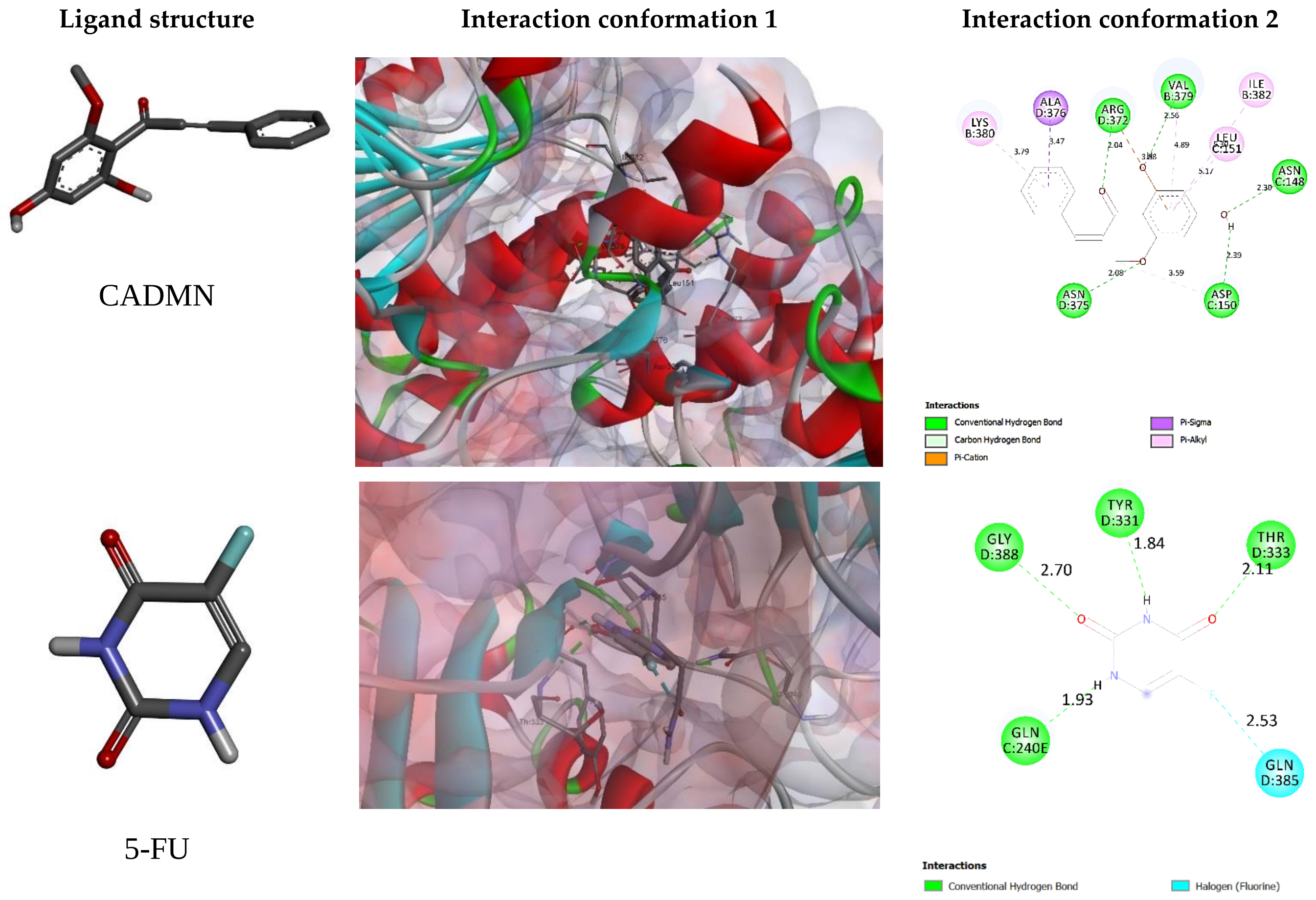

Figure S7. The interactions between caspase 9 and ligands (CADMN and 5-FU)

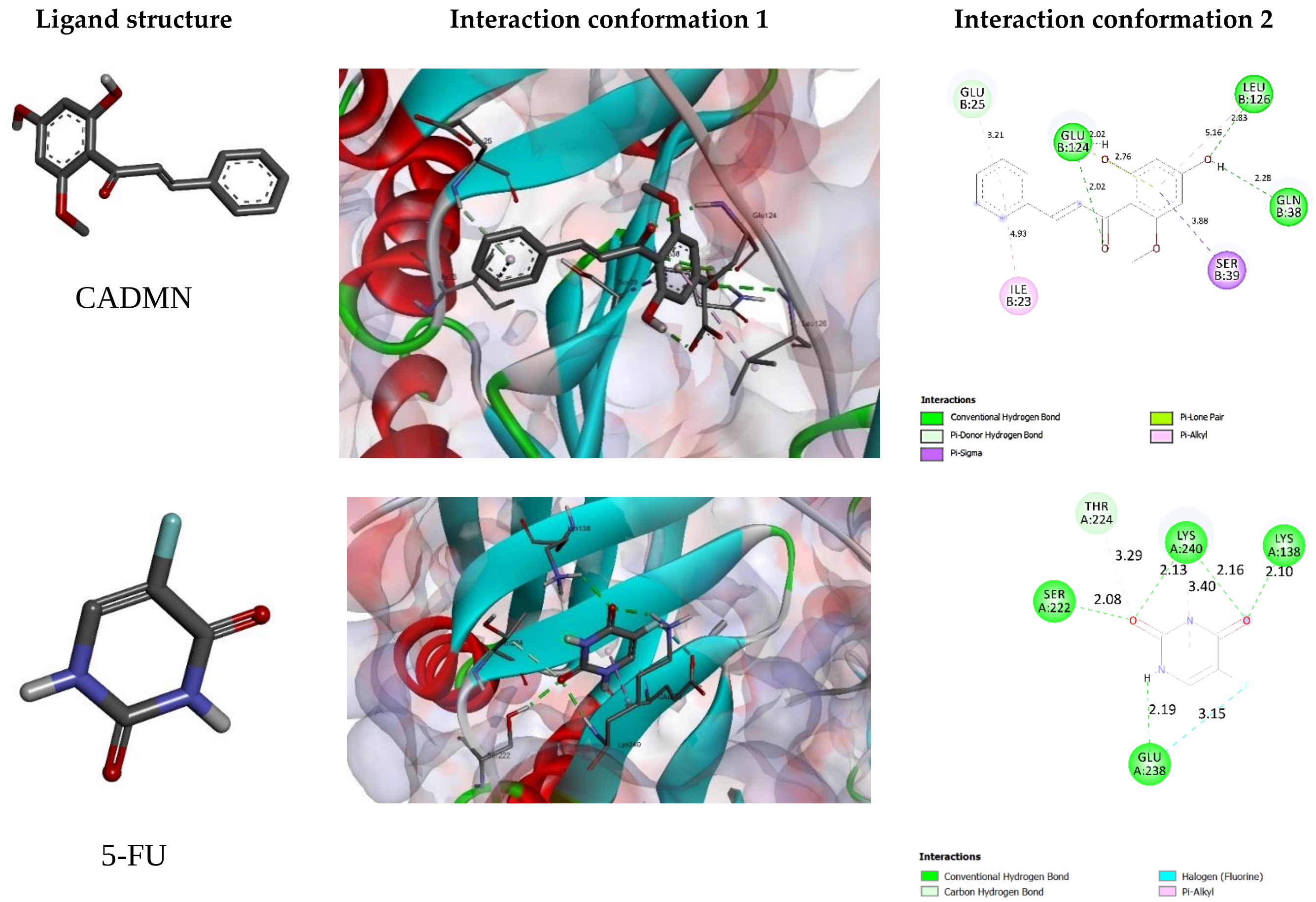

Figure S8. The interactions between PCNA and ligands (CADMN and 5-FU)

Ligand structure **Interaction conformation 1** **Interaction conformation 2**

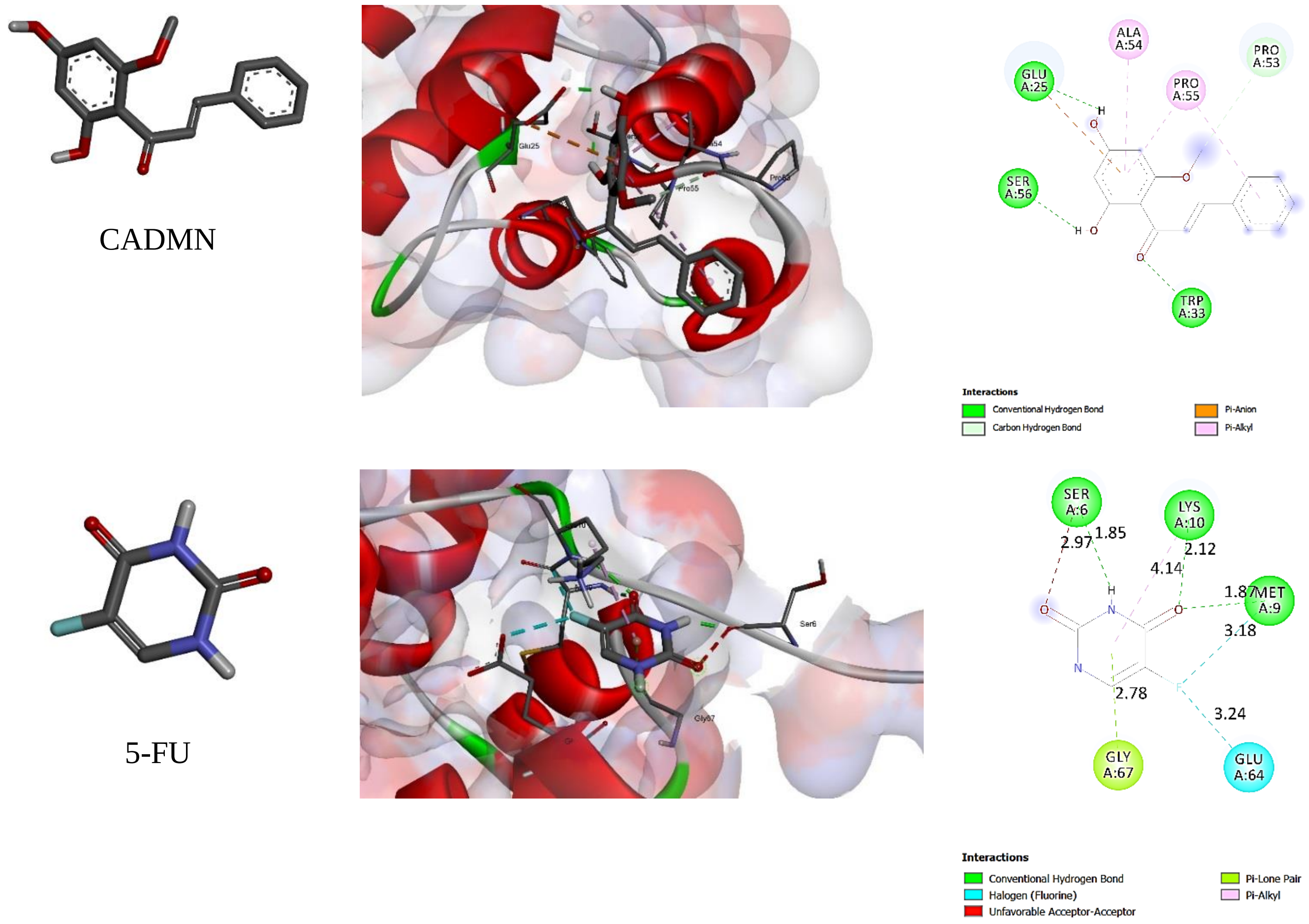

Figure S9. The interactions between NF-κB and ligands (CADMN and 5-FU)

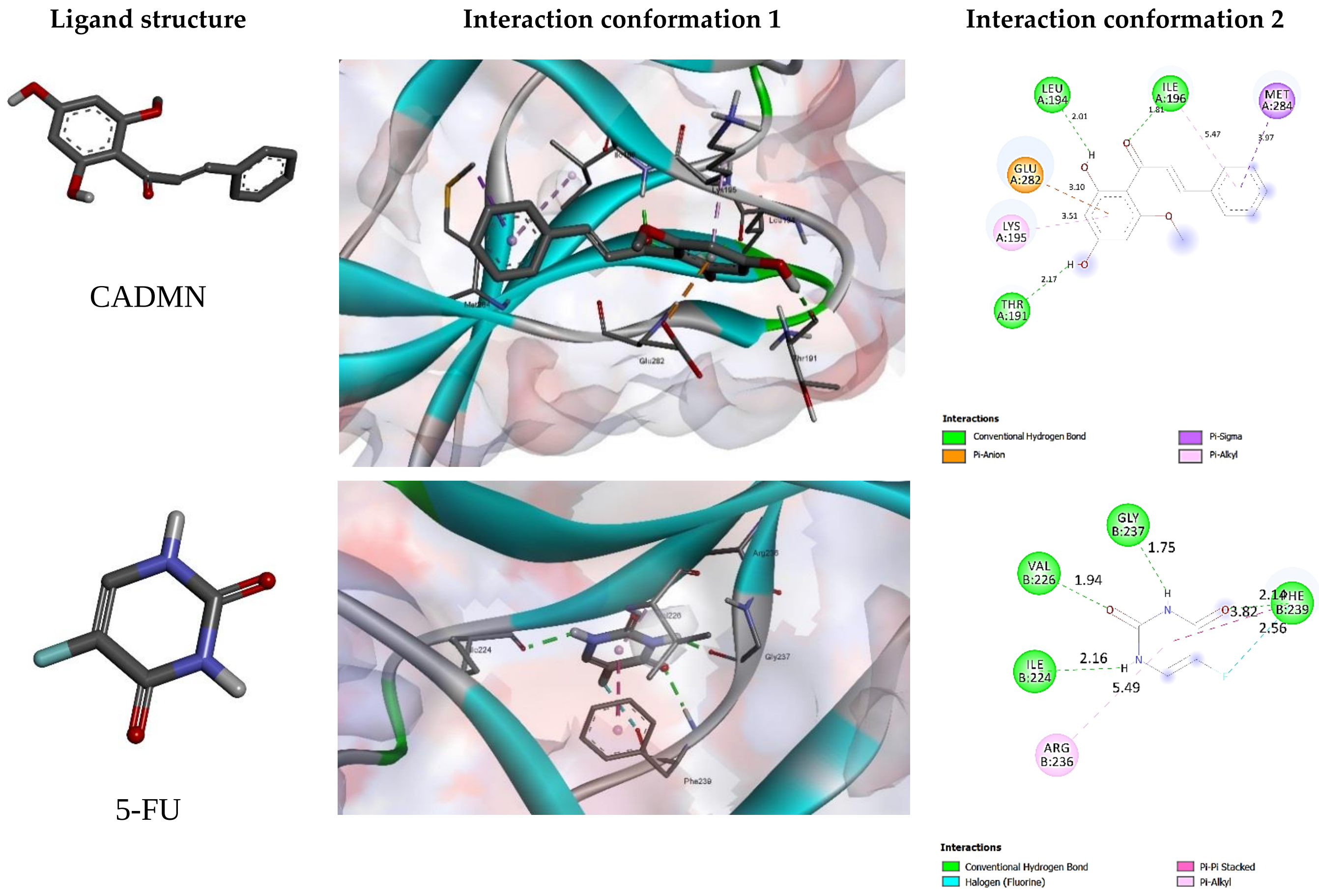

Figure S10. The interactions between NF-κB-p65 and ligands (CADMN and 5-FU)

Ligand structure	Interaction conformation 1	Interaction conformation 2

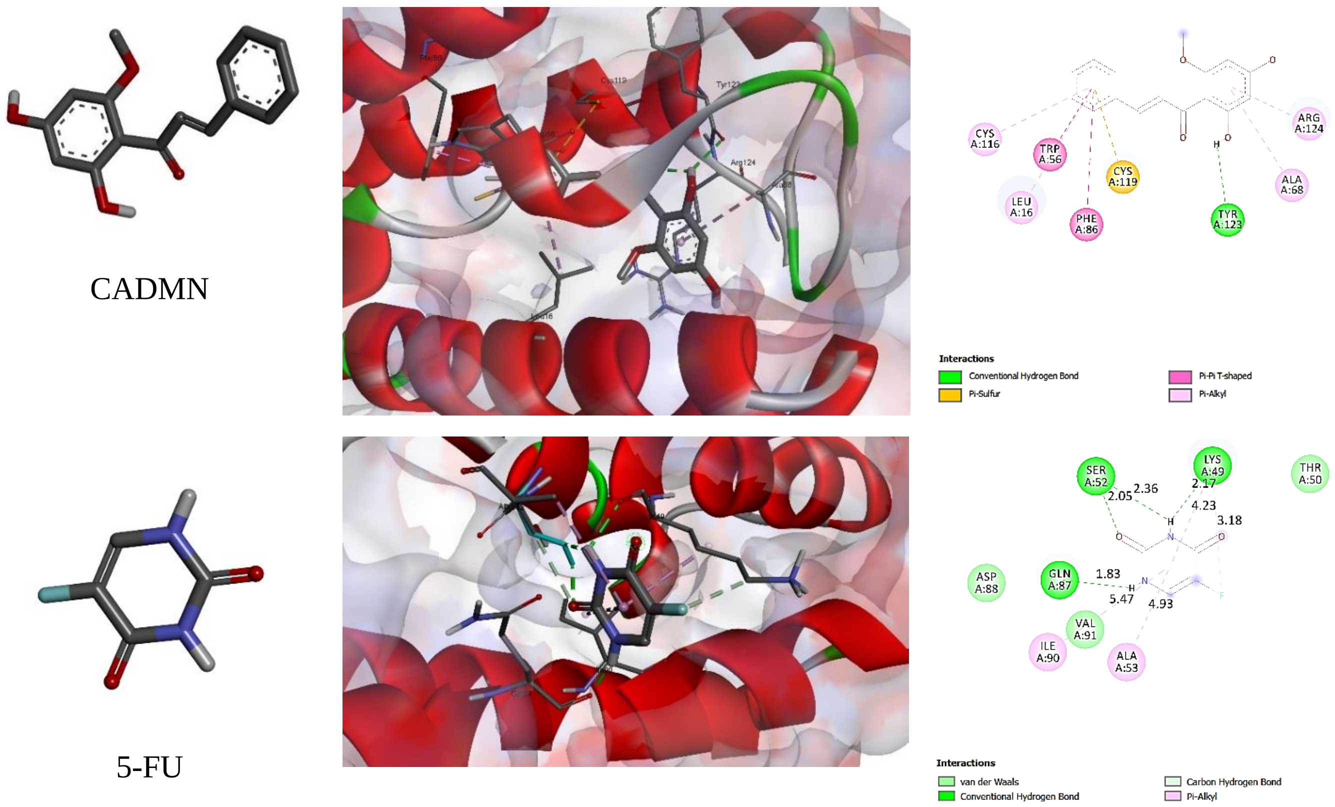

Figure S11. The interactions between cytochrome C and ligands (CADMN and 5-FU)
